# Effects of Exercise in Immersive Virtual Environments on Cortical Neural Oscillations and Mental State

**DOI:** 10.1155/2015/523250

**Published:** 2015-08-20

**Authors:** Tobias Vogt, Rainer Herpers, Christopher D. Askew, David Scherfgen, Heiko K. Strüder, Stefan Schneider

**Affiliations:** ^1^Institute of Movement and Neurosciences, German Sport University Cologne, Am Sportpark Müngersdorf 6, 50933 Cologne, Germany; ^2^Institute of Visual Computing and Department of Computer Science, Bonn-Rhein-Sieg University of Applied Sciences, Grantham-Allee 20, 53757 Sankt Augustin, Germany; ^3^Department of Computer Science and Engineering, York University, 4700 Keele Street, Toronto, ON, Canada M3J 1P3; ^4^Faculty of Computer Science, University of New Brunswick, 550 Windsor Street, Fredericton, NB, Canada E3B 5A3; ^5^School of Health and Sport Sciences, Faculty for Science, Health, Education and Engineering, University of the Sunshine Coast, Maroochydore DC, QLD 4558, Australia

## Abstract

Virtual reality environments are increasingly being used to encourage individuals to exercise more regularly, including as part of treatment those with mental health or neurological disorders. The success of virtual environments likely depends on whether a sense of presence can be established, where participants become fully immersed in the virtual environment. Exposure to virtual environments is associated with physiological responses, including cortical activation changes. Whether the addition of a real exercise within a virtual environment alters sense of presence perception, or the accompanying physiological changes, is not known. In a randomized and controlled study design, moderate-intensity Exercise (i.e., self-paced cycling) and No-Exercise (i.e., automatic propulsion) trials were performed within three levels of virtual environment exposure. Each trial was 5 minutes in duration and was followed by posttrial assessments of heart rate, perceived sense of presence, EEG, and mental state. Changes in psychological strain and physical state were generally mirrored by neural activation patterns. Furthermore, these changes indicated that exercise augments the demands of virtual environment exposures and this likely contributed to an enhanced sense of presence.

## 1. Introduction

While the beneficial effects of exercise for physical and mental health are well recognized, average physical activity is below recommended levels, and it remains a challenge to support the adoption of active behaviours [1]. Virtual reality environments are increasingly being used to encourage individuals to exercise more regularly [2], for the prevention of sedentary lifestyles among relatively healthy individuals, including, and as part of treatment, in those with mental health or neurological disorders [3].

When exercising in virtual environments for the benefit of mental health, there is a need to consider the theoretical sense of presence [3]. Sense of presence is defined as a perceived feeling of immersion when exposed to a virtual environment; it is perfected when one is completely unaware of their real surroundings [4–7]. Beyond subjective assessments to explore a sense of presence, there is increasing reliance on more objective physiological measurements, for example, heart rate monitoring [8]. Only a few studies have investigated the neural responses that underlie the sense of presence perception. There has been a particular focus on the neural activation patterns that accompany and may regulate the sense of presence during the process of habituation to virtual environments [9, 10]. Recently, frontal brain regions (i.e., dorsolateral prefrontal cortex) were identified to form a key node for a sense of presence network (SPN; [11]). Participants who are highly engaged and most attentive during imagination and movement representation studies show strong SPN activation [11]. Electroencephalography (EEG) frequency analyses have also been used to explore the neural activation patterns associated with sense of presence perception. Alterations in alpha oscillations, particularly over the frontoparietal brain regions, have been associated with perceived sense of presence [12]. Notwithstanding continuous debates on inconsistent findings, alpha and beta oscillations are also associated with mental state, for example, physical and psychological strain [10, 13]. It has been shown that sense of presence perception depends largely on the characteristics of the virtual environment and differs with screen size, duration of exposure, and the realism of the presentation [10, 14–16]. Whether the addition of a real exercise (e.g., cycling on an ergometer) within an immersive virtual environment alters sense of presence perception, or the accompanying changes in neural activation patterns or mental state, is not known.

In healthy participants, the beneficial effects of exercise on mental state and mental well-being are reflected by neurophysiological and behavioural adaptations [17, 18]. These effects of exercise on mental well-being are suggested to be largely dependent on the volume or “dose” of exercise [19, 20] and are also influenced by a preference bias towards self-paced (moderate-intensity) exercise [21]. Studies of brain activity and the alterations in alpha and beta oscillations with exercise suggest the presence of a general model of cortical arousal (MCA; [22]). According to this, a reduction in alpha activity and an increase in beta activity reflect cortical arousal; and the reverse is associated with relaxed cortical states that may be recorded immediately after self-paced (moderate-intensity) exercise [20]. The relationship between exercise-induced changes in mental state and neural activation patterns may also be described on the basis of the transient hypofrontality theory (THT; [23]). Transient hypofrontality would suggest that the EEG changes that accompany exercise reflect redistribution of limited cortical resources away from less required brain regions (e.g., decreased activity in frontal brain regions) towards more required brain regions (e.g., increased activity in motor regions). Thus, based upon the dominant role of frontal brain regions in mental state, this exercise-induced redistribution is in favour of well-being (i.e., decreased activity in frontal brain regions as a refreshment of mental state capacity). Transient hypofrontality is particularly evident in response to self-paced (moderate-intensity) exercise, and similar responses are seen across a diverse range of participants including younger and older participants and those with and without a mental impairment [24–26]. With this, combining traditional interpretations of alterations in EEG frequency bands (i.e., MCA) with the latest understanding of cortical redistribution (i.e., THT) seems reasonable. It is not known whether these same neural responses occur during exercise or movement representation in a virtual environment, and this is important to establish if virtual environments are to be used to facilitate exercise as a therapy for mental well-being.

Therefore, the objective of this study was to investigate the interactive effects of virtual environment exposure and exercise on physiological and perceptual responses in healthy adults. Specifically, we aimed to examine the influence of moderate-intensity exercise (i.e., self-paced cycling), compared with movement representation, within three levels of virtual environment exposure (a complex three-screen mode, a simple one-screen mode, and a no-screen Control) on cortical neural oscillations (i.e., frontal alpha and beta activity), perceived sense of presence, and mental state (i.e., perceived physical state, motivational state, and psychological strain).

## 2. Materials and Methods

### 2.1. Participants

Participants were eighteen healthy volunteers (7 females, 11 males) with no known history of neurological or musculoskeletal disorders (age: 28.78 ± 5.19 years; height: 177.50 ± 10.15 cm; weight: 75.44 ± 13.23 kg). Participants considered themselves recreationally active with no particular experience of being exposed to virtual environments. All participants gave written informed consent to participate. This study was approved by the institutional Human Research Ethics Committee and was conducted in accordance with the Declaration of Helsinki.

### 2.2. Experimental Procedures

**Figure 1:**
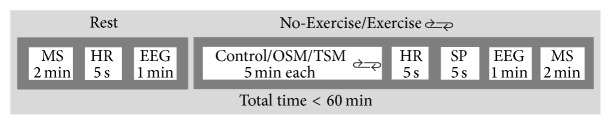
Randomized (indicated by rolling arrows) and controlled* study design*. Exercise and No-Exercise trials were conducted within three levels of virtual environment exposure (Control; OSM: one-screen mode; TSM: three-screen mode). Mental state (MS), heart rate (HR), sense of presence (SP), and EEG measurements were made at rest and following each trial.

Participants attended a single experimental test session, following familiarisation with the cycle ergometer and the test environment on the same day. Experimental procedures were arranged in a randomized and controlled study design. After baseline measurements, Exercise or No-Exercise trials were conducted within three levels of virtual environment exposure. Each trial was 5 minutes in duration and was followed by posttrial assessments of heart rate, perceived sense of presence, and EEG as well as mental state (Figure 1).

#### 2.2.1. Virtual Environment Exposure

**Figure 2:**
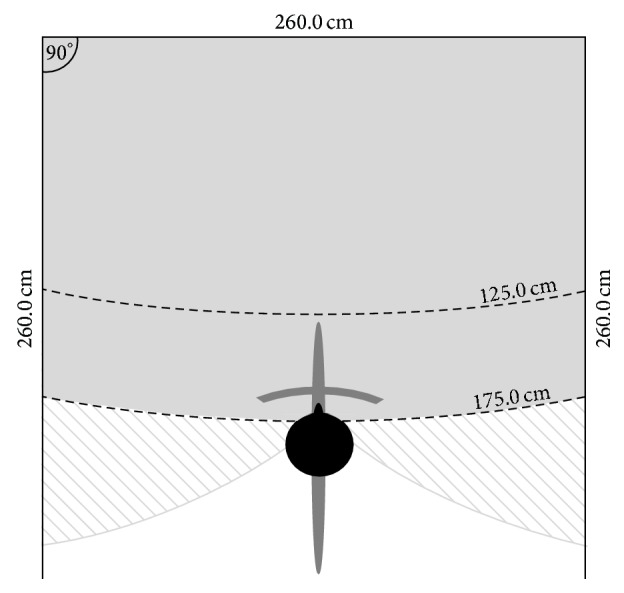
Overhead view of the* Immersion Square system*, including three screens (bold black lines, each 260.0 cm wide) with a participant seated on a cycling ergometer (black head and darker grey schematic). The participant's visual field (depicted by the lighter grey zone and if the head turns left/right the dashed lighter grey zone) was centred between 125.0 and 175.0 cm from the front screen.

An Immersion Square system (Bonn-Rhein-Sieg University of Applied Sciences; [27]) was used to generate immersive 3D content. Back projection (8 GB RAM, AMD Radeon HD 5800 Series) on three screens (260.0 cm width, 195.0 cm height) provided 4200 × 1050 pixel resolution (each 1400 × 1050 pixels). Screens were arranged in a square surrounding the participant's visual field (Figure 2). Screen configuration allowed for three levels of virtual environment exposure: (1) one-screen mode (OSM) with only the front screen switched on, (2) three-screen mode (TSM) with all three screens switched on, and (3) Control with all screens switched off.

#### 2.2.2. No-Exercise versus Exercise Trials

During each trial, participants were seated on a FIVIS bicycle simulator (Bonn-Rhein-Sieg University of Applied Sciences; [4]). During both the OSM and TSM trials, 3D content was identical and comprised an endless two-lane road (straight) in an urban setting [28]. The 3D video display was synchronised with the actions of the bicycle, including steering (i.e., handlebar movement), velocity (i.e., pedal cadence and torque), and braking (i.e., backpedal or handbrake). With respect to dose [19, 20] and preference [21], Exercise trials were performed at a self-paced moderate intensity that was defined by consistent heart rate monitoring and verbal instructions prior to each Exercise trial (i.e., “Please pace your pedalling consistently to meet your perceived moderate intensity”). During No-Exercise trials, participants sat on the cycle ergometer without moving and the virtual bicycle (3D video) travelled the course at a fixed virtual speed of 22.0 km/h.

### 2.3. Data Processing

#### 2.3.1. Heart Rate Monitoring

Heart rate was monitored using a Polar RCX5 portable heart rate monitor (Polar Electro Oy, Finland). Average heart rate (bpm) at rest and during the period immediately after each trial was used for analysis.

#### 2.3.2. Sense of Presence Perception

Perceived sense of presence was assessed immediately after each trial using a simple verbal query/response-questionnaire [28, 29]. Participants were asked, “Without hesitating, how strongly do you feel connected to the virtual environment at this moment?” Responses were anchored to an 11-step scale from 0 (not at all) to 10 (totally).

#### 2.3.3. Assessment of Mental State

Mental state was assessed using the MoodMeter that consists of Bodyfinder and Feelfinder modules. The Bodyfinder module is sensitive to short-term mood alterations and has been developed to determine current perceived physical state (PEPS) based on the subdomains of physical energy, physical fitness, physical flexibility, and physical health. This module has been used and validated in various biomedical settings, including exercise physiology and internal medicine studies [30]. The Feelfinder module comprises a shortened version of the EZ-scale (“Eigenzustandsskala”; [31]). In comparison to other psychological adjective scales (e.g., POMS) the EZ-scale assesses motivational state (MOT), in addition to the commonly assessed psychological strain (PSYCH). This study used a 16-item EZ-scale [31], comprising eight subdomains to generate assessments of MOT (self-confidence, willingness to seek contact, social acceptance, and readiness for strain) and PSYCH (relaxation, positive mood, calmness, and recovery).

For this study, the MoodMeter was configured with 32 adjectives (16 PEPS, 8 MOT, and 8 PSYCH) that were stored and presented to participants on a handheld Axim X50 pocket PC (Dell, USA) in a quasi-random sequence. Upon the presentation of each adjective, participants were asked to indicate how well that adjective described their current physical or mental state by selecting one of six options from 0 (not at all) to 5 (totally). The response time for each adjective was limited to five seconds so as to discourage rational deliberation, and this time was shown with a progress bar at the bottom of the screen. With this, the MoodMeter was specifically designed to detect short-term alterations that are of particular relevance for exercise-related research. On each occasion, completion of the mood assessment (all 32 adjectives) took less than two minutes. For further details on the development and operation of the MoodMeter, please refer to Kleinert [30].

#### 2.3.4. Recording of Cortical Neural Oscillations

To record cortical neural oscillations, BrainVision Recorder 1.20.0701 together with a portable actiCAP system was used (BrainProducts GmbH, Germany). A permeable-to-air EEG-cap that is adapted to individual head size was mounted up to 10 minutes before testing. The EEG-cap consisted of 64 Ag/AgCl electrodes, arranged in the international 10:20 system [32]: Fp1, Fp2, AF7, AF3, AF4, AF8, F7, F5, F3, F1, Fz, F2, F4, F6, F8, FT9, FT7, FC5, FC3, FC1, FC2, FC4, FC6, FT8, FT10, T7, C5, C3, C1, Cz, C2, C4, C6, T8, TP9, TP7, CP5, CP3, CP1, CPz, CP2, CP4, CP6, TP8, TP10, P7, P5, P3, P1, Pz, P2, P4, P6, P8, PO7, PO3, POz, PO4, PO8, O1, Oz, O2, and PO10. FCz (reference) and AFz (ground) were added and PO9 served as horizontal electrooculogram (EOG) to detect eye movements. SuperVisc electrode gel (EasyCap GmbH, Germany) was added to each electrode to optimize conductivity. Distances between electrodes were >25 mm to avoid bridging (though not measured, perspiration was not noticeably profuse in any participant). A 60-second resting EEG was recorded (sampling rate of 500 Hz) with eyes closed, while seated on the cycle ergometer before (rest) and after each trial.

Analogue EEG data were amplified and digitally converted for analyses using BrainVision Analyser 2.1.0.327 (BrainProducts GmbH, Germany). Low and high cutoff filter frequencies ranged between 7.5 and 45.0 Hz (time constant 0.02 seconds, 48 dB/octave). Based on the EOG, Gratton's standard ocular correction [33] was performed to reduce eye-moving artifacts. After segmentation and an automatic artifact rejection (gradient < 35 *μ*V, min/max amplitudes ± 100 *μ*V), a minimum of twelve 4-second segments remained for Fast Fourier Transformation (spectral analysis: resolution at 0.24 Hz, Hanning window of 10%). Averaged segments were pooled into frontal (Fp1, Fp2, AF7, AF3, AF4, AF8, F7, F5, F3, F1, Fz, F2, F4, F6, F8, FT9, FT7, FC5, FC3, FC1, FC2, FC4, FC6, FT8, and FT10), central (C5, C3, C1, Cz, C2, C4, C6, CP5, CP3, CP1, CPz, CP2, CP4, and CP6), parietal (P7, P5, P3, P1, Pz, P2, P4, P6, and P8), and occipital (PO7, PO3, POz, PO4, PO8, O1, Oz, O2, and PO10) electrode sites before exporting prominent frequency bands alpha (7.5–12.5 Hz) and beta (12.5–35.0 Hz) as mean activity in *μ*V. For statistical comparisons, frontal, central, parietal, and occipital pools were each referenced to a global pool (export of all respective other electrode sites).

### 2.4. Statistical Analyses

All statistical analyses were performed using the software Statistica 7.1 (StatSoft, Tulsa, USA).

Repeated-measures analysis of variance (ANOVA) was used to detect condition effects and interactions between exercise (Exercise, No-Exercise) and virtual environment exposure (Control, OSM, and TSM) for heart rate, cortical neural oscillations (EEG: alpha, beta), and mental state (PEPS, MOT, and PSYCH). Fisher's least significant difference (LSD) was applied* post hoc* where interactions and main effects were identified. Friedman's ANOVA followed by Wilcoxon paired samples test was used to identify changes in the perceived sense of presence. Analysis of covariance (ANCOVA) was computed to determine possible effects of gender. Possible correlations between cortical neural oscillations (EEG: alpha, beta), mental state (PEPS, MOT, and PSYCH), sense of presence, and heart rate in both No-Exercise and Exercise trials were determined using Pearson's correlation coefficient. Data (*n* = 18) in figures are presented as mean ± confidence interval (0.95) and as mean ± standard error of mean in the text and tables. Significance was set at *p* < 0.05.

## 3. Results

### 3.1. Heart Rate

Heart rates showed significant increases from rest to No-Exercise and Exercise trials (*F*_(3,51)_ = 39.63, *p* < 0.05), and these changes were consistent across the three levels of virtual environment exposure (Table 1). ANCOVA revealed no significant effect of gender on heart rate (*λ* = 0.58,* F*_(7,9)_ = 0.92, *p* > 0.05).

### 3.2. Sense of Presence

Sense of presence increased from No-Exercise to Exercise trials, with increasing levels of virtual environment exposure from Control to OSM and TSM (Chi^2^_(18,5)_ = 83.98, *p* < 0.05; Table 2). ANCOVA revealed no significant gender effect for perceived sense of presence (*λ* = 0.52,* F*_(6,10)_ = 1.52, *p* > 0.05).

### 3.3. Mental State

There were significant main effects where mental state differed between Exercise and No-Exercise trials and between the different levels of virtual environment exposure (*F*_(4,68)_ = 3.37, *p* < 0.05).* Post hoc* analysis revealed increases in PEPS and PSYCH from Control to OSM and TSM during the Exercise trial, whereas MOT increased from Control to OSM and TSM in the No-Exercise trial (Table 3). There was no significant gender effect (ANCOVA: *λ* = 0.52,* F*_(15,1)_ = 1.22, *p* > 0.05).

### 3.4. Cortical Neural Oscillations

#### 3.4.1. Alpha Activity

**Figure 3:**
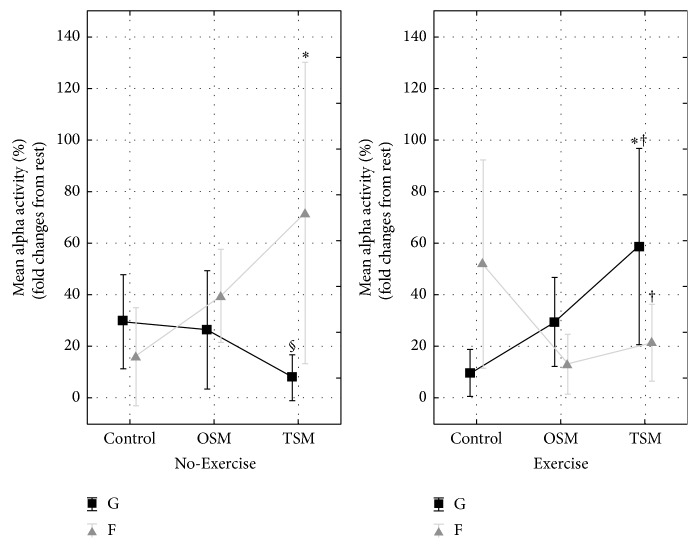
*Mean alpha activity* (7.5–12.5 Hz), expressed as relative (%) changes over global (G; black lines) and frontal (F; grey lines) electrode sites. Mean EEG activity was measured before (rest) and following No-Exercise (automatic drive) and Exercise (moderate cycling) trials within three levels of virtual environment exposure (Control; OSM: one-screen mode; TSM: three-screen mode). *∗* indicates a significant difference compared with Control (*p* < 0.05); † indicates difference between Exercise and No-Exercise trials (*p* < 0.05); § indicates difference between F and G (*p* < 0.05). Data are mean ± 0.95 CI (*n* = 18).

Frontal alpha activity showed significant differences between No-Exercise and Exercise trials across the various virtual environment exposures (*F*_(2,34)_ = 5.90, *p* < 0.05).* Post hoc* analysis revealed frontal alpha activity increased from Control to TSM during the No-Exercise condition, whereas the respective global alpha activity did not change with levels in virtual environment exposure. The reverse was shown during the Exercise trial where the respective global alpha activity increased from Control to TSM, whereas frontal alpha activity did not change significantly with levels in virtual environment exposure (Figure 3). ANCOVA revealed no significant effect of gender for alpha activity (*λ* = 0.12,* F*_(12,4)_ = 2.45, *p* > 0.05).

Central (*F*_(2,34)_ = 1.60, *p* > 0.05), parietal (*F*_(2,34)_ = 2.16, *p* > 0.05), and occipital (*F*_(2,34)_ = 2.86, *p* > 0.05) alpha activity revealed no significant changes compared to their respective global alpha activity.

#### 3.4.2. Beta Activity

**Figure 4:**
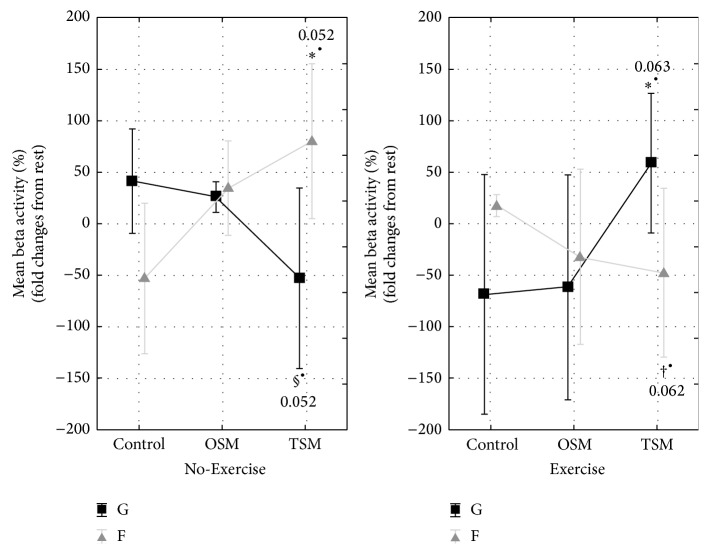
*Mean beta activity* (12.5–35.0 Hz), expressed as relative change (%) from rest over global (G; black lines) and frontal (F; grey lines) electrode sites. Mean EEG activity was measured before (rest) and following No-Exercise (automatic drive) and Exercise (moderate cycling) trials within three levels of virtual environment exposure (Control; OSM: one-screen mode; TSM: three-screen mode). *∗* ^∙^ indicates a trend compared with Control (*p* < 0.1); †^∙^ indicates difference between Exercise and No-Exercise trials (*p* < 0.1); §^∙^ indicates difference between F and G (*p* < 0.05). Data are mean ± 0.95 CI (*n* = 18).

Frontal beta activity showed significant differences between No-Exercise and Exercise trials during the different levels of virtual environment exposure (*F*_(2,34)_ = 5.11, *p* < 0.05).* Post hoc* analysis revealed that, during the No-Exercise trial, frontal beta activity increased from Control to TSM by trend, whereas the respective global beta activity did not change significantly. These findings were reversed during the Exercise trial, where the respective global beta activity increased from Control to TSM by trend, and frontal beta activity did not change significantly (Figure 4). There was no significant gender effect (ANCOVA: *λ* = 0.15,* F*_(12,4)_ = 1.92, *p* > 0.05).

Central (*F*_(2,34)_ = 1.59, *p* > 0.05), parietal (*F*_(2,34)_ = 0.78, *p* > 0.05), and occipital (*F*_(2,34)_ = 1.55, *p* > 0.05) beta activity revealed no significant changes compared to their respective global beta activity.

### 3.5. Correlations

For the No-Exercise trial, positive correlations could be obtained between cortical neural oscillations and mental state as well as between cortical neural oscillations and heart rate (Table 4).

For the Exercise trial, a positive correlation could be obtained between mental state and heart rate (Table 5).

## 4. Discussion

This study aimed to investigate the interactive effects of virtual environment exposure and exercise on neurophysiological and perceptual responses in healthy adults. We verified that exposure to increasing levels of virtual environment exposure led to increases in perceived sense of presence, and a key finding was that this change in perception was further enhanced by the inclusion of exercise. Measures of mental state responded to increasing levels of virtual environment exposure, and the addition of exercise led to increases in physical state (PEPS) and psychological strain (PSYCH) and a reduction in motivational state (MOT). The addition of exercise to the virtual environment had a significant influence on the EEG responses, with a shift from strong frontal alpha and beta activity responses during the No-Exercise condition to strong global alpha and beta responses when exercise was added to the virtual environment exposure.

Perceived sense of presence increased with increasing levels of virtual environment exposure, from Control to one-screen mode (OSM) and three-screen mode (TSM), and in each condition sense of presence was further amplified with the addition of exercise. Though not correlated, these changes in sense of presence were underlined by changes in heart rate. The increases in heart rate during the No-Exercise condition are consistent with previous studies [8], and collectively these findings support the use of heart rate monitoring as an objective physiological measure that may reflect sense of presence during exposure to virtual environments.

There was a significant increase in PEPS and PSYCH during exercise in the virtual environments compared with the No-Exercise condition. The increase in PEPS (perceived physical state) reflects the increased heart rate and effort during exercise, and such a response is well documented in the literature [17–20]. The strong PSYCH (psychological strain) response to exercise in the virtual environments appears to be at odds with evidence of the beneficial and “calming” effects of exercise [20], although exercise alone in the present study (during Control) did in fact lead to a slight (not significant) reduction in PSYCH. The increase in PSYCH during exercise in the virtual environment was accompanied by a reduction in motivation (MOT). Though surprising, it seems reasonable that exercising in virtual environments may lead to less motivation, relative to an increasing virtual environment exposure (i.e., from Control to OSM to TSM) that is coexistent with an increase in PSYCH (e.g., increased discomfort). Also, it has previously been suggested that virtual reality increases the psychological demands of an environment, and these demands increase with increased duration of exposure [10]. Our findings suggest that the addition of exercise increases the demands of a virtual environment and this results in a pronounced psychological strain. In contrast, it appears that less demanding virtual environments (i.e., without exercise) result in a stronger motivational state [14], thus, in line with the above suggested less motivation while exercising in the virtual environment.

The increase in global beta activity after exercise in the virtual environment is consistent with the hypothesis of a generalised model of cortical arousal (MCA). According to the MCA, the increase in beta activity is consistent with cortical arousal and has previously been associated with the incidence of “cybersickness,” akin to motion sickness [10], and this corresponds with the elevated psychological strain and the decreased motivation during this trial. Rather than a corresponding decrease in alpha activity, as would be expected according to the MCA, global alpha activity also increased during exercise in the virtual environment. This is suggestive of a relaxed cortical state [34], which is consistent with the low motivational state in the current trial, but at odds with the elevated psychological strain. Assessment of cortical current density allows for the localisation of neural changes to specific regions of the brain. The transient hypofrontality theory (THT) reflects redistribution of cortical activity away from the frontal regions [23, 25], thus allowing for greater cortical resources to maintain mental state. While there were significant increases in frontal cortical neural oscillations in the virtual environment during the No-Exercise condition, the addition of exercise led to reductions in both alpha and beta frontal activity, relative to global activity. This is consistent with the THT and presumably is driven largely by the increase in physical and psychological strain.

From a sense of presence perspective, the neural activation patterns are in line with previous neuroimaging studies, suggesting that frontal brain regions form a key sense of presence node [11]. Though documented in a nonexercise fMRI study (i.e., rollercoaster scenarios), Baumgartner et al. [9] suggest lesser cortical activation in the dorsolateral prefrontal cortex accompanied by a stronger sense of presence perception. In addition, it is well accepted that frontoparietal brain regions are strongly involved in movement control. Even the imagination of movements or motor representation is likely to shape the sense of presence perception in virtual environments [35]. With this and previous evidence that postural changes enhance sense of presence in virtual environments [36], it seems reasonable that the inclusion of real exercise (i.e., self-paced cycling on an ergometer) similarly fosters and possibly intensifies sense of presence perception compared to motor representation (i.e., automatic drive). This is underlined by increased sense of presence perception from No-Exercise to Exercise trials as well as from Control to OSM and TSM.

### 4.1. Limitations

Despite plausible findings, we are aware that the present study is limited by a rather small number of participants. Additionally, lack of data pertaining to exercise intensity (e.g., watt/rpm, VO_2_) makes it difficult to verify the standardisation of exercise conditions; however, the heart rate responded as would be expected to further support the moderate exercise definition, which is mainly owing to available infrastructure.

## 5. Conclusions

The primary aim of this study was to examine cortical neural oscillations and related alterations in mental state in response to exercise in immersive virtual environments compared to movement representation. Changes in psychological strain and physical state were generally mirrored by neural activation patterns of frontal and global alpha and beta activity. Furthermore, these changes are likely to indicate, based on the model of cortical arousal and, in particular, the transient hypofrontality theory, that exercise augments the demands of virtual environment exposures and this possibly contributes to enhanced sense of presence.

## Figures and Tables

**Table 1:** Heart rate [bpm].

	Rest	Control	OSM	TSM
No-Exercise	75.22 ± 1.79	90.17 ± 2.70^*∗*†^	90.11 ± 3.02^*∗*†^	90.22 ± 3.07^*∗*†^
Exercise		122.89 ± 5.21^*∗*^	126.00 ± 5.14^*∗*^	121.89 ± 5.16^*∗*^

Heart rate (bpm) was measured before (rest) and following No-Exercise (automatic drive) and Exercise (moderate cycling) trials within three levels of virtual environment exposure (Control; OSM: one-screen mode; TSM: three-screen mode). *∗* indicates a significant difference compared with rest (*p* < 0.05); † indicates a significant difference between Exercise and No-Exercise trials (*p* < 0.05). Data are mean ± SEM (*n* = 18).

**Table 2:** Perceived sense of presence [absolute values].

	Control	OSM	TSM
No-Exercise	0.22 ± 0.13	3.44 ± 0.41^*∗*†^	5.44 ± 0.46^*∗*§†^
Exercise	0.06 ± 0.06	4.67 ± 0.41^*∗*^	7.22 ± 0.34^*∗*§^

Perceived sense of presence, expressed as absolute values (scale: 0 = no sense of presence at all, 10 = full sense of presence), was measured before (rest) and following No-Exercise (automatic drive) and Exercise (moderate cycling) trials within three levels of virtual environment exposure (Control; OSM: one-screen mode; TSM: three-screen mode). *∗* indicates a significant difference compared with Control (*p* < 0.05); ^§^difference compared with OSM (*p* < 0.05); ^†^difference between Exercise and No-Exercise trials (*p* < 0.05). Data are mean ± SEM (*n* = 18).

**Table 3:** Mental state [%].

	PEPS	MOT	PSYCH
No-Exercise			
Control	8.82 ± 6.56	−1.25 ± 5.55	−2.68 ± 6.16
OSM	−1.46 ± 6.39^†^	26.09 ± 8.22^*∗*†•^	6.77 ± 5.64
TSM	3.85 ± 6.65	22.94 ± 7.08^*∗*^	3.47 ± 8.54^†^
Exercise			
Control	−2.07 ± 4.76	10.46 ± 7.98	−1.55 ± 4.86
OSM	26.09 ± 8.22^*∗*^	3.02 ± 7.26	21.73 ± 10.63^*∗*^
TSM	22.94 ± 7.08^*∗*^	5.27 ± 6.80	34.76 ± 8.66^*∗*^

Mental state, expressed as relative (%) changes from rest for perceived physical state (PEPS), motivational state (MOT), and psychological strain (PSYCH). Mental state assessments were measured before (rest) and following No-Exercise (automatic drive) and Exercise (moderate cycling) trials within three levels of virtual environment exposure (Control; OSM: one-screen mode; TSM: three-screen mode). *∗* indicates a significant difference compared with Control (*p* < 0.05); ^†^difference between Exercise and No-Exercise trials (*p* < 0.05); ^†•^trend between Exercise and No-Exercise trials (*p* < 0.1). Data are mean ± SEM (*n* = 18).

**Table 4:** 

	No-Exercise								
	Alpha^G^	Alpha^F^	Beta^G^	Beta^F^	PEPS	MOT	PSYCH	SP	HR
Alpha^G^					*r* ^2^ = 0.02	*r* ^2^ = 0.04	*r* ^2^ = 0.02	*r* ^2^ = 0.01	*r* ^2^ = 0.09^*∗*•^
Alpha^F^					*r* ^2^ = 0.02	*r* ^2^ = 0.15^*∗*^	*r* ^2^ = 0.12^*∗*•^	*r* ^2^ = 0.00	*r* ^2^ = 0.17^*∗*^
Beta^G^					*r* ^2^ = 0.09^*∗*^	*r* ^2^ = 0.05^*∗*^	*r* ^2^ = 0.11^*∗*^	*r* ^2^ = 0.00	*r* ^2^ = 0.00
Beta^F^					*r* ^2^ = 0.00	*r* ^2^ = 0.01	*r* ^2^ = 0.01	*r* ^2^ = 0.01	*r* ^2^ = 0.00
PEPS	*r* ^2^ = 0.02	*r* ^2^ = 0.02	*r* ^2^ = 0.09^*∗*^	*r* ^2^ = 0.00				*r* ^2^ = 0.01	*r* ^2^ = 0.00
MOT	*r* ^2^ = 0.04	*r* ^2^ = 0.15^*∗*^	*r* ^2^ = 0.05^*∗*^	*r* ^2^ = 0.01				*r* ^2^ = 0.01	*r* ^2^ = 0.06^*∗*•^
PSYCH	*r* ^2^ = 0.02	*r* ^2^ = 0.12^*∗*•^	*r* ^2^ = 0.11^*∗*^	*r* ^2^ = 0.01				*r* ^2^ = 0.01	*r* ^2^ = 0.01
SP	*r* ^2^ = 0.01	*r* ^2^ = 0.00	*r* ^2^ = 0.00	*r* ^2^ = 0.01	*r* ^2^ = 0.01	*r* ^2^ = 0.01	*r* ^2^ = 0.01		*r* ^2^ = 0.00
HR	*r* ^2^ = 0.09^*∗*•^	*r* ^2^ = 0.17^*∗*^	*r* ^2^ = 0.00	*r* ^2^ = 0.00	*r* ^2^ = 0.00	*r* ^2^ = 0.06^*∗*•^	*r* ^2^ = 0.01	*r* ^2^ = 0.00	

Coefficient of positive correlations between cortical neural oscillations (alpha^G^: global mean alpha activity; alpha^F^: frontal mean alpha activity; beta^G^: global mean beta activity; beta^F^: frontal mean beta activity), mental state (PEPS: perceived physical state; MOT: motivational state; PSYCH: psychological strain), sense of presence (SP), and heart rate (HR) for No-Exercise trial. *∗*• indicates a trend (*p* < 0.1); *∗* indicates a significant correlation (*p* < 0.05).

**Table 5:** 

	Exercise								
	Alpha^G^	Alpha^F^	Beta^G^	Beta^F^	PEPS	MOT	PSYCH	SP	HR
Alpha^G^					*r* ^2^ = 0.06^*∗*•^	*r* ^2^ = 0.01	*r* ^2^ = 0.00	*r* ^2^ = 0.02	*r* ^2^ = 0.00
Alpha^F^					*r* ^2^ = 0.01	*r* ^2^ = 0.00	*r* ^2^ = 0.00	*r* ^2^ = 0.01	*r* ^2^ = 0.05^*∗*•^
Beta^G^					*r* ^2^ = 0.04	*r* ^2^ = 0.02	*r* ^2^ = 0.01	*r* ^2^ = 0.00	*r* ^2^ = 0.00
Beta^F^					*r* ^2^ = 0.01	*r* ^2^ = 0.03	*r* ^2^ = 0.02	*r* ^2^ = 0.00	*r* ^2^ = 0.03
PEPS	*r* ^2^ = 0.06^*∗*•^	*r* ^2^ = 0.01	*r* ^2^ = 0.04	*r* ^2^ = 0.01				*r* ^2^ = 0.00	*r* ^2^ = 0.02
MOT	*r* ^2^ = 0.01	*r* ^2^ = 0.00	*r* ^2^ = 0.02	*r* ^2^ = 0.03				*r* ^2^ = 0.00	*r* ^2^ = 0.08^*∗*^
PSYCH	*r* ^2^ = 0.00	*r* ^2^ = 0.00	*r* ^2^ = 0.01	*r* ^2^ = 0.02				*r* ^2^ = 0.00	*r* ^2^ = 0.03
SP	*r* ^2^ = 0.02	*r* ^2^ = 0.01	*r* ^2^ = 0.00	*r* ^2^ = 0.00	*r* ^2^ = 0.00	*r* ^2^ = 0.00	*r* ^2^ = 0.10		*r* ^2^ = 0.00
HR	*r* ^2^ = 0.00	*r* ^2^ = 0.05^*∗*•^	*r* ^2^ = 0.00	*r* ^2^ = 0.03	*r* ^2^ = 0.02	*r* ^2^ = 0.08^*∗*^	*r* ^2^ = 0.03	*r* ^2^ = 0.00	

Coefficient of positive correlations between cortical neural oscillations (alpha^G^: global mean alpha activity; alpha^F^: frontal mean alpha activity; beta^G^: global mean beta activity; beta^F^: frontal mean beta activity), mental state (PEPS: perceived physical state; MOT: motivational state; PSYCH: psychological strain), sense of presence (SP), and heart rate (HR) for Exercise trial. *∗*• indicates a trend (*p* < 0.1); *∗* indicates a significant correlation (*p* < 0.05).
